# Editorial: Advancing intervertebral disc degeneration research: focus on annulus fibrosus and pain management

**DOI:** 10.3389/fspor.2026.1788795

**Published:** 2026-02-04

**Authors:** Jiawei Lu, Lutian Yao, Feng Wang, Haiyue Zu, Chris Halling Dreyer

**Affiliations:** 1 Department of Spine Surgery, Shanghai East Hospital, School of Medicine, Tongji University, Shanghai, China; 2 Department of Orthopaedic Surgery, First Hospital of China Medical University, China Medical University, Shenyang, China; 3 Department of Orthopaedic Surgery, First Hospital of Soochow University, School of Medicine, Soochow University, Suzhou, China; 4 Department of Orthopaedic Surgery and Traumatology, CORI - Centre for Orthopaedic Research and Innovation, Central and West Zealand Hospital, Slagelse, Denmark; 5 Institute of Regional Health Research - IRS, Faculty of Health Science, University of Southern Denmark, Odense, Denmark

**Keywords:** annulus fibrosus (AF), discogenic pain, intervertebral disc degeneration (IDD), lumbar disc herniation (LDH), regenerative therapies

Intervertebral disc degeneration (IVDD) constitutes a major source of chronic low back pain and functional limitation worldwide, substantially impairing physical activity, sports performance, and quality of life across diverse populations. The annulus fibrosus (AF), the robust outer fibrous component of the intervertebral disc, is indispensable for preserving disc integrity, resisting tensile and shear forces, and containing the nucleus pulposus. Progressive AF pathology—including matrix breakdown, micro-fissures, inflammation, neoinnervation, and reduced biomechanical competence—serves as a pivotal driver of disc herniation, segmental instability, and persistent discogenic pain. Although important progress has been made in elucidating the molecular, cellular, and biomechanical mechanisms of AF degeneration, significant challenges persist: inconsistent treatment efficacy, high recurrence rates after intervention, incomplete understanding of pain generation pathways, and limited options for true structural restoration.

This Research Topic was specifically designed to address these unmet needs by gathering the latest scientific and clinical advances in intervertebral disc degeneration, with a dedicated focus on annulus fibrosus pathology and its intimate relationship with chronic pain and physical function. The Research Topic aims to promote interdisciplinary dialogue, integrate innovative therapeutic strategies with rehabilitation principles, and bridge foundational research with real-world clinical application. By emphasizing emerging interventions—ranging from regenerative biologics and minimally invasive structural repair to integrative conservative therapies and patient-centered education—the Research Topic seeks not only to improve pain control but also to enhance disc preservation or repair, ultimately enabling affected individuals to maintain or regain active lifestyles more effectively and sustainably.

The six articles published within this Research Topic provide valuable, complementary contributions that advance these objectives:

A large cross-sectional survey evaluated knowledge, attitudes, and practices (KAP) among 395 patients diagnosed with lumbar disc herniation. While knowledge levels were generally inadequate, positive attitudes and self-management behaviors were common; importantly, higher knowledge directly and indirectly predicted improved practices and lower disability (ODI). These findings strongly support the integration of structured patient education programs to enhance long-term self-care and clinical outcomes.

A prospective, randomized, double-blind, placebo-controlled trial investigated the combination of Haitongpi Formula Gel Paste (a contemporary formulation rooted in classical Traditional Chinese Medicine) with Qing dynasty three-movement manipulation in patients with lumbar disc herniation. The active intervention produced significantly greater and more sustained reductions in pain intensity (VAS) and disability (ODI) compared with placebo, alongside an excellent safety profile, illustrating the promise of integrative conservative strategies in managing disc-related symptoms.

A comprehensive systematic review and meta-analysis synthesized evidence from eight randomized controlled trials (total *n* = 1,744) on intradiscal medical ozone injections for lumbosacral pain. Ozone therapy demonstrated statistically and clinically meaningful short-term superiority over conventional treatments in both pain relief (VAS) and functional improvement (ODI), reinforcing its role as an attractive, minimally invasive option while calling for additional long-term follow-up studies.

An instructive case report documented the first described instance of Anaerococcus vaginalis spondylodiscitis, successfully identified through nanopore targeted sequencing despite repeatedly negative conventional cultures, and effectively managed with targeted antimicrobial therapy following surgical intervention. This work highlights the transformative potential of next-generation sequencing technologies in diagnosing uncommon infectious contributors to disc degeneration and pain.

A head-to-head prospective randomized controlled trial compared intradiscal platelet-rich plasma (PRP) injection with methylene blue in patients suffering from discogenic low back pain. PRP treatment resulted in significantly better outcomes across pain (VAS), lumbar function (JOA), and objective imaging markers of disc health (apparent diffusion coefficient and Pfirrmann grade), providing encouraging evidence for the regenerative potential of biologics in modulating the degenerative disc microenvironment.

Finally, a dedicated clinical study assessed annulus fibrosus suture (AFS) as an adjunct to percutaneous transforaminal endoscopic discectomy (PTED) in obese patients with single-level lumbar disc herniation. The combined procedure yielded robust improvements in leg pain (VAS-LP), disability (ODI), and neurological function (JOA) at one-year follow-up, with no cases of recurrent herniation or need for secondary open surgery. Although back pain relief was comparatively more limited, the addition of AFS meaningfully reduced re-herniation risk in this biomechanically vulnerable population.

Taken together, these contributions reflect a growing multidisciplinary effort to tackle annulus fibrosus-centered challenges through precision diagnostics, regenerative therapies, minimally invasive structural repair, integrative conservative modalities, and empowered patient self-management. They collectively move the field closer to more durable, function-preserving solutions for intervertebral disc degeneration and associated pain.

We express our deep appreciation to the authors, reviewers, and the Frontiers in editorial team for their dedication and expertise in bringing this Research Topic to fruition. As Topic Editors (Jiawei Lu, Lutian Yao, Feng Wang, Haiyue Zu, and Chris Halling Dreyer), we trust that the insights presented here will catalyze further innovation, collaboration, and progress toward improved care for individuals living with disc degeneration.

